# Behavioral Intention Promotes Generalized Reciprocity: Evidence From the Dictator Game

**DOI:** 10.3389/fpsyg.2020.00772

**Published:** 2020-04-30

**Authors:** Zhongqiang Sun, Chuyuan Ye, Zhihui He, Wenjun Yu

**Affiliations:** ^1^Department of Psychology, Ningbo University, Ningbo, China; ^2^Center of Group Behavior and Social Psychological Service, Ningbo University, Ningbo, China; ^3^Business School, Ningbo University, Ningbo, China; ^4^Academy of Neuroeconomics and Neuromanagement, Ningbo University, Ningbo, China

**Keywords:** generalized reciprocity, intention, generosity, greed, the dictator game

## Abstract

Generalized reciprocity is the phenomenon that individuals treat others in the same way that others treated them in the past. Besides the behavioral outcomes, whether intention information also manipulates generalized reciprocal behavior remains unclear. By conducting two rounds of the dictator game, the current research investigated the influence from the dictator’s intention on the receiver’s following resource allocation performance. In the games, in order to allocate, either tokens in Experiment 1 or jobs in Experiment 2, a general tendency was shown to treat others better if one was generously treated than greedily treated. Regarding the intentionality, participants who received a generous offer (vs. greedy offer) from another person (i.e., intentional) would perform more generously to another person. However, if the offer was randomly given by a computer program (i.e., unintentional), the way in which one was being treated previously, became somewhat irrelevant to the participants’ generalized reciprocal behaviors. Those findings verified the influence of the manipulation of intention on generalized reciprocity, and provided enlightenment for promoting friendly social interactions.

## Introduction

As an important part of the evolution of cooperation, reciprocal behaviors are ubiquitous in human society. Reciprocity manifests in several ways. Direct reciprocity, in the form of tit-for-tat, happens in the interaction between two individuals (Trivers, 1971); in indirect reciprocity, the agent treats A similarly to how A treated B (Nowak and Sigmund, 2005). More recently, under the circumstances of extensive one-shot interactions between two strangers in society currently, another reciprocity, called “generalized reciprocity” (Pfeiffer et al., 2005), has received increasing attention from researchers. Generalized reciprocity^1^ refers to a situation whereby a person who had been treated positively or negatively by others in the past delivers a treatment following the same pattern to someone else, commonly referred to as “paying it forward” (Gray et al., 2014). As a principal component of moral codes, generalized reciprocity might induce a series of chain reactions from the initial biased behavior (e.g., Nowak and Sigmund, 2005; Liu et al., 2015). Evolutionary studies have confirmed that combined with certain behavioral strategies, generalized reciprocity could bring the benefits of generating cooperation behaviors and promoting interpersonal communication (Nowak and Roch, 2007; Rankin and Taborsky, 2009). Other research fields, such as psychology (Alvarez and van Leeuwen, 2015; Halali et al., 2017), cognitive neuroscience (Watanabe et al., 2014), and management (Simpson et al., 2018), have also expressed keen interest in the phenomenon of generalized reciprocity.

According to whether the delivered reciprocal behavior indeed assists the receiver, generalized reciprocity may be classified as positive or negative. Positive generalized reciprocity refers to a transmission of prosocial behaviors when a person receives support from others (Herne et al., 2013; Horita et al., 2016), or more generally follows the principle of “you help me, I help someone else.” Findings from a scenario simulation experiment demonstrated that participants who had received other’s help were more willing to answer a stranger’s questionnaires than those who had not (Bartlett and DeSteno, 2006). DeSteno et al. (2010) also found a comparatively generous performance in token allocation tasks with participants who had been positively treated (vs. having been negatively treated), manifesting as a prosocial behavior transmission. Conversely, poor treatment in the past might lead to a similar selfish behavior toward other people, reflecting negative generalized reciprocity (e.g., Zitek et al., 2010; Gray et al., 2014). Compared to individuals recalling common things, for instance, individuals recalling unfair experiences were much more likely to refuse another’s assistance (Zitek et al., 2010). Recent research has provided more direct evidence through two rounds of the “dictator game” (Gray et al., 2014). In each round of this game, two participants are given a sum of monetary units in common, and one of them (named the “dictator”) can decide to take some of the monetary units for himself/herself and leave the rest for the other (named the “receiver”). The receiver must unconditionally accept the dictator’s offer and cannot provide feedback to the dictator. The dictator can make the offer completely as (s)he wishes without having to take the receiver’s reciprocal behavior into consideration. The results showed that participants generously (greedily) treated in the first round would give more money to a third person as dictator in the second round.

Given the above, previous literature mainly focused on the influence of consequences of earlier actions on generalized reciprocal behaviors, yet little is known about this issue with respect to the individual’s cognitive processing. What kinds of actions might promote or inhibit generalized reciprocal behavior, and, furthermore, does the action information affect positive and negative reciprocal behaviors in similar ways? Studies on reciprocity have shown that one’s behavior intention, in addition to action consequences, also plays a role (e.g., Dufwenberg and Kirchsteiger, 2004). Rabin (1993) proposed that individuals exhibit direct reciprocity based on their judgment of the fairness of an action, which originates from the perception of others’ intention. A general theory of reciprocity (Falk and Fischbacher, 2006) states that reciprocal behavior is a response to others’ kindness, and behavioral outcome and intention are the two main determinants of kindness perception. In a direct reciprocity study (Falk et al., 2008), for instance, participants were told to be a receiver and a dictator in two sequential rounds of ultimate games. When participants realized that the offer in the first round was given by a real person (vs. throwing the dice), direct reciprocal behavior was enhanced, manifesting as a positive correlation between the money allocated in the first round and the amount of their rewards/punishment given to the benefactor in the second round. However, if it was a computer program that provided the preceding offer, this correlation sharply weakened. Other reciprocity research has verified this wide and prominent impact of intention (Stanca et al., 2011; Chao, 2018), and also found it to develop from a relatively young age. Children as early as three years of age manifested generosity if they had received benefit from intentional others (Vaish et al., 2018).

As described above, manipulation of intention has been well established in the direct and indirect reciprocity literature. One might speculate that there is an analogous effect of intention in generalized reciprocity. Nevertheless, since generalized reciprocal behavior involves a new stranger who has not been interacted with previously, simply inferring from (in)direct reciprocity could be misleading. Specifically, in instances of direct and indirect reciprocity, people actually interact with the initial actor twice, so behavioral intention is a keyfactor to evaluate the actor’s personality traits, to predict future performance, and finally, to determine whether to cooperate with him/her (Orhun, 2018). This might not be so for generalized reciprocity, however. When the next interacting stranger is no longer the previous one, the former perceived intention may become somewhat irrelevant. Thus, current research aims to explore whether intention information matters in generalized reciprocal behavior.

To our knowledge, only one study thus far has examined the influence of intention on generalized reciprocity (Herne et al., 2013). In that study, researchers manipulated intention by setting the dictator as a person or a computer program, but failed to find the above influence. Instead of evaluating the other’s offer received in the previous round, the participant was required to deal with all allocation probabilities. Herne and colleagues stated that their non-significant results might have been due to this measurement of the dependence variable, which forced participants to undertake strategic deliberation of each allocation option while the dictator’s intention might have been overlooked, so the results did not reflect the effect of intention.

Therefore, current research has emphasized the role of intention in generalized reciprocal behavior. To implement reciprocity, two rounds of dictator games were sequentially performed in both experiments (e.g., Gray et al., 2014; Strang et al., 2016), whereby a participant played the role as the receiver unconditionally receiving the other’s allocation offer, and then acted as a dictator. Following the manipulation of intention (intentional human vs. unintentional computer program) in Herne et al. (2013), participants in the current study would receive only one kind of allocation offer in the first round (we thus used a between-subjects design), leading them to focus on the intentional information. The authenticity of the current study was ensured by the participants’ real engagement in anonymous dictator games, and the robustness was enhanced by using two kinds of resource carrier – tokens in Experiment 1 and workload in Experiment 2. We hypothesized that intention would reinforce generalized reciprocal behaviors (including both positive and negative ones), and that this effect might be weakened in unintentional situations.

## Experiment 1

### Materials and Methods

#### Participants

In all, 128 graduate and undergraduate students (68 females; mean age 19.67 ± 0.75 years, ranging from 18 to 21 years old) volunteered to participate in the experiment. All participants had normal or corrected-to-normal vision, and provided informed consent in compliance with the Declaration of Helsinki before the experiment. Twelve participants who misunderstood the instructions or reported being aware of the disguised manipulation after the experiment were excluded from further analysis.

#### Design and Procedure

We used E-Prime 2.0 for presentation and response acquisition. The procedures and study design were approved by the Research Ethics Board of Department of Psychology in Ningbo University. A 2 (Intentional vs. Unintentional) × 2 (Greedy vs. Generous) between-subjects design was adopted in the current study.

Before the experiment, the participant was first labeled B and told to conduct two rounds of LAN-based money allocation tasks with two other players A and C. Communication among the three players was restricted to a greeting when they first met in the laboratory. To ensure anonymity, we asked all players whether they knew each other already, and the answers were all negative. In order to fix the participant’s role as a reciprocal behavior deliverer in two rounds of money allocation tasks, all tasks were operated by an offline program in the formal experiment, and both players A and C were experimenters disguised as players who did not actually perform the tasks.

**FIGURE 1:**
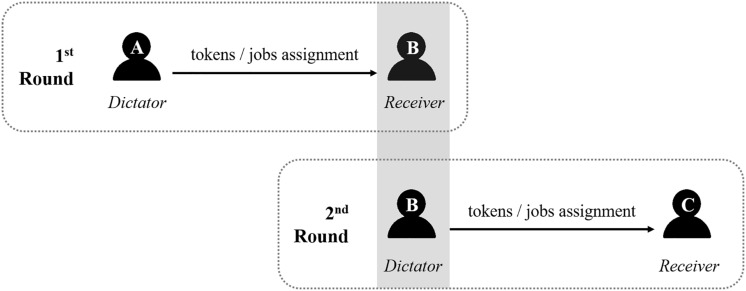
The real role assignment of the two-round dictator game for the participant in Experiments 1 and 2.

After number assignment, all three players (A and C in disguise) were instructed together by another experimenter regarding the procedure. We adopted the dictator game as the main task, and the instructions for all players were as follows: “You are going to play two rounds of a money-sharing task with two other players via a LAN-based program. At the beginning of each round, two of you will be paired, then each will be randomly assigned to be dictator or receiver by the program; the other needs to wait for the next round. If you are assigned to be dictator, you will receive 100 tokens in total as initial funding; please choose an allocation plan for you both (intentional condition)/wait for the program to randomly choose an allocation plan for you both (unintentional condition); if you are the receiver, please wait for the dictator/program’s decision and accept it unconditionally. After a short rest, the second round will begin. When all rounds finish, you will receive payment corresponding to your final tokens.” To ensure general reciprocity, the participant performed two rounds of the dictator game in the real manipulation, in the first round always as a receiver and the second round always as a dictator, as illustrated in Figure 1.

All players were directed to three different rooms after the instruction, and only the participant actually performed the following formal tasks. The participant was seated at a distance of 70 cm from the monitor and was required to complete two rounds in total on the computer. At the beginning of the first round, an assignment page was presented stating that the role assignment procedure was run by the program. About 5 s later, the participant was shown as being paired as the receiver with player A in this round. Then (s)he was shown a silhouette of player A (dictator) and two options of money allocation for the dictator on the screen, including a greedy allocation (70 for the dictator and 30 for the receiver) and a generous allocation (30 for the dictator and 70 for the receiver). After a waiting page displaying “the dictator is making the choice.” (intentional condition)/“the program is randomly allocating.” (unintentional condition) for 4–8 s, participants in the greedy condition would see that the dictator/program chose the greedy allocation plan and that they received 30 tokens, while those in the generous condition would see that the dictator/program chose the generous allocation plan and they received 70 tokens. After a rest of several seconds, the second round (i.e., final round as instructed) started. The same role assignment page was presented for about 5 s, followed by a page telling the participant that he/she was paired with player C and assigned to be the dictator. The program offered the participant 100 tokens and asked him/her to allocate it to player C as (s)he wished.

After completing the experiment, participants were asked to report whether they were aware of the disguised manipulation, as well as to make a guess of the current experimental objective. Anyone who answered affirmatively was labeled an outlier and excluded from the final analysis.

#### Data Analysis

The number of tokens allocated to player C in the final round was recorded as the dependent variable. Repeated-measures ANOVA was conducted to analyze the generalized reciprocal behavior, with the Intentionality and Initial treatment as the variables. Significant interaction (*p* < 0.05) was followed by simple-effect analyses.

### Results

**FIGURE 2:**
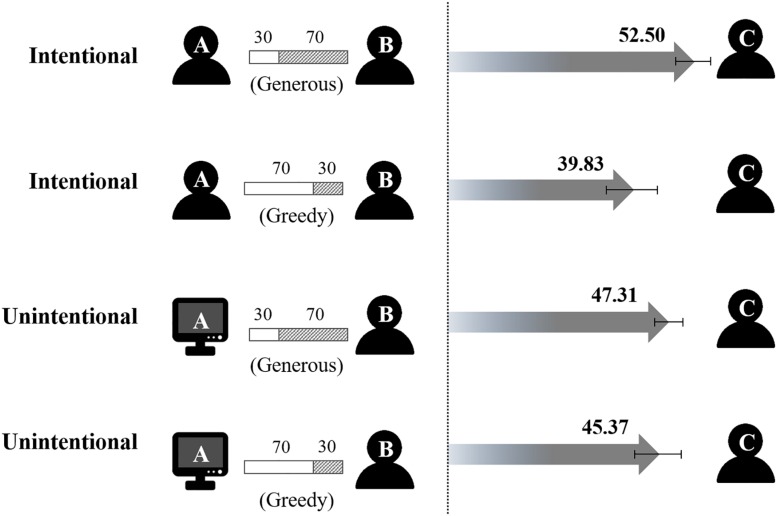
The manipulation of the treatments in the first round of dictator game and results in Experiment 1. The number above gray arrow represents the average number of tokens allocated to player C among total 100 tokens, and the error bar represents one S.D.

The allocation amounts of tokens are depicted in Figure 2. A 2 (Intentionality: intentional, unintentional) × 2 (Initial treatment: greedy, generous) between-subjects ANOVA yielded a significant main effect of initial treatment, *F*(1, 112) = 20.117, *p* < 0.001, η*_*p*_*^2^ = 0.152. Participants in the generous condition (*M* ± *S.D.*, 49.86 ± 7.19) allocated more tokens to player C than those in the greedy condition did (42.56 ± 10.62). An interaction between intentionality and initial treatment was also significant, *F*(1, 112) = 10.88, *p* = 0.001, η*_*p*_*^2^ = 0.089. The main effect of intentionality was non-significant, *F*(1, 112) = 0.012, *p* = 0.913, η*_*p*_*^2^ < 0.001.

More specifically, one-way ANOVA for each initial treatment condition revealed that, when treated generously in the previous round of the dictator game, participants in the intentional condition (52.50 ± 7.39; vs. unintentional condition, 47.31 ± 6.08) paid forward more tokens, *F*(1, 55) = 0.012, *p* = 0.005, η*_*p*_*^2^ = 0.133; when being treated greedily, on the contrary, those in the intentional condition (39.83 ± 10.89; vs. unintentional condition, 45.37 ± 9.89) gave much less to player C, *F*(1, 57) = 0.012, *p* = 0.044, η*_*p*_*^2^ = 0.069. In addition, one-way ANOVA was also applied to each intentionality condition. When the choice was made by player A in the first round (i.e., intentional), the allocation amount of tokens for player C was higher in the generous than the greedy condition, *F*(1, 56) = 26.474, *p* < 0.001, η*_*p*_*^2^ = 0.321. However, the difference diminished when the initial treatment was randomly made by the program (i.e., unintentional), *F*(1, 56) = 0.823, *p* = 0.368, η*_*p*_*^2^ = 0.014.

## Experiment 2

One might argue that the virtual tokens in Experiment 1 would weaken participants’ engagement in the allocation tasks. To enhance authenticity, Experiment 2 explored generalized reciprocal behavior by using a “token” more commonly encountered in real life – workload. Two types of job, interesting and dull, were adopted to replace the above tokens. Participants first performed both jobs to familiarize themselves with them, and then allocated those jobs in the formal experiment.

### Materials and Methods

#### Participants

In total, 126 graduate and undergraduate students (86 females; mean age 19.78 ± 0.90 years, ranging from 18 to 24 years old) volunteered to participate in the experiment. All participants had normal or corrected-to-normal vision, and provided informed consent in compliance with the Declaration of Helsinki before the experiment. Ten participants who misunderstood the instructions or reported being aware of the disguised manipulation after the experiment were excluded from further analysis.

#### Design and Procedure

The general procedures and between-subjects design were almost the same as in Experiment 1, which had been approved by the Research Ethics Board of Department of Psychology in Ningbo University.

However, two kinds of job, interesting and dull, replaced the tokens in Experiment 1. The interesting job was a word-association game, in which the participant was asked to associate content with three given words. Any content was acceptable and no limit was set for the length. The dull job required the participant to count the vowels in an English paragraph, and the job was finished when the count number was correct. The participant performed both jobs before the experiment. The interesting job lasted half a minute, and the dull one took about 3 min.

In the first round of the formal experiment, the dictator was given 10 interesting and 10 dull jobs. The receiver needed to wait for the dictator (intention condition)/program’s (unintentional condition) choice between the following two options of job allocation: (1) seven interesting and three dull jobs for the dictator, and the remaining three interesting and seven dull jobs for the receiver (greedy option); and (2) three interesting and seven dull jobs for the dictator, and the remaining seven interesting and three dull jobs for the receiver (generous option). In the first round, the participant was always assigned to be the receiver, and those in the greedy condition saw the dictator/program choose the greedy option, while those in the generous condition saw the dictator/program choose the generous option. In the second round, the participant was always the dictator, and was asked to allocate part of all 20 new jobs (including 10 interesting and 10 dull jobs) to player C as (s)he wished, so as to assign 10 jobs to each of them.

#### Data Analysis

The number of interesting jobs for player C was recorded as the dependent variable. Repeated-measures ANOVA was conducted to analyze the generalized reciprocal behavior, with the Intentionality and Initial treatment as the variables. Significant interaction (*p* < 0.05) was followed by simple-effect analyses.

### Results

**FIGURE 3:**
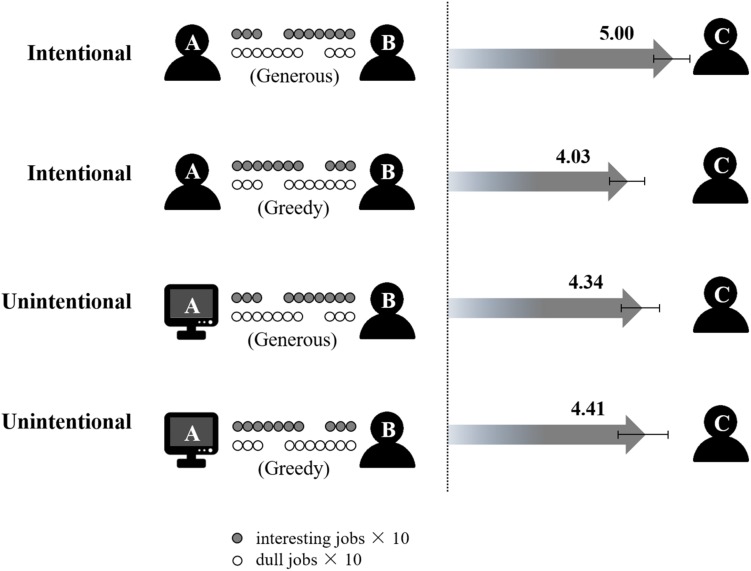
The manipulation of the treatment in the first round of dictator game and the results in Experiment 2. The number above gray arrow is the average number of interesting jobs for player C among all 10 interesting jobs, and the error bar represents one S.D. The solid circle represents one interesting job, and the hollow circle represents one dull job.

As shown in Figure 3, we found a significant main effect of treatment, *F*(1, 112) = 7.208, *p* = 0.008, η*_*p*_*^2^ = 0.06. *Post-hoc* analysis showed that generous treatment (4.67 ± 0.12) was followed by a similar more generous assignment of interesting jobs to player C than was greedy treatment (4.22 ± 0.12). The interaction between intentionality and initial treatment was also significant, *F*(1, 112) = 9.596, *p* = 0.002, η*_*p*_*^2^ = 0.079, while the main effect of intentionality was non-significant, *F*(1, 112) = 0.682, *p* = 0.411, η*_*p*_*^2^ = 0.006.

Simple effect analysis for this interaction effect replicated a similar pattern as in Experiment 1. More specifically, when previously treated generously, participants in the intentional condition (5.00 ± 0.80) tended to assign more interesting jobs to player C than those in the unintentional condition (4.34 ± 0.86), *F*(1, 56) = 9.041, *p* = 0.004, η*_*p*_*^2^ = 0.139, while the situation was reversed when (s)he was treated greedily (intentional, 4.03 ± 0.78, vs. unintentional, 4.41 ± 1.12), *F*(1, 56) = 2.247, *p* = 0.140, η*_*p*_*^2^ = 0.039. Additionally, participants in the intentional condition assigned more interesting jobs to the receiver when treated generously than greedily in the previous round, *F*(1, 56) = 21.649, *p* < 0.001, η*_*p*_*^2^ = 0.279, while no difference was found for participants in the unintentional condition between the generous and greedy conditions, *F*(1, 56) = 0.069, *p* = 0.793, η*_*p*_*^2^ = 0.001.

## Discussion

By sequentially assigning the participant to be the receiver and then the dictator in two rounds of the dictator game, the current research explored the influence of intention on generalized reciprocity. In the task of allocating either tokens (Experiment 1) or jobs (Experiment 2), participants presented a more generous reciprocal behavior in the intentional than the unintentional condition when they were treated generously previously.; When the participants were treated greedily before, they manifested a similar greedier reciprocal behavior in the intentional condition than unintentional condition. The above results are the first to reveal an evident influence of intention on generalized reciprocal behaviors.

Both experiments confirmed that current initial treatments could efficiently manipulate reciprocity. In line with a previous study (Gray et al., 2014; Horita et al., 2016), the allocation amount to the next person in the greedy treatment condition was significantly smaller than in the generous treatment condition. The findings also guarantee the reliability of the current paradigm.

More importantly, this is the first study showing the key role of human intention on generalized reciprocal behavior; that is, the unique existence of intention in humans could shed light on the effect of previous treatment on future behavior toward someone else. In the current experiments, if a person was treated generously by a person (vs. a computer program), (s)he would share more tokens or interesting jobs with other strangers; contrariwise, if treated greedily by a person (vs. a computer program), participants would assign fewer tokens or interesting jobs to someone else. These results suggest that regardless of treatment type (i.e., generous or greedy), intention always has an amplified impact on generalized reciprocity. Beyond that, such influence of intention on generalized reciprocity provides supporting evidence for previous findings in many other fields, including moral judgment (e.g., Cushman et al., 2013; Gan et al., 2016) and direct reciprocity (e.g., Vaish et al., 2018), and extends our knowledge of the effect of prior intention to generalized reciprocal behaviors.

Another interesting finding was that the behavioral transmission patterns of generous and greedy behaviors differed only when the offer was made as intentional behavior. Unlike direct reciprocity, in which the reciprocal behavior still more or less exists (weaker for unintentional offers than intentional ones, but not eliminated) if (s)he had previously been treated unintentionally (e.g., Falk et al., 2003; Charness, 2004), the current results demonstrate that the offer from an unintentional program did not affect the participant’s assignment pattern toward others at all. This is a breakthrough finding from the aspect of generalized reciprocity. As our interactions with strangers are expanding and being facilitated in step with technological development, it is thus of great importance to clarify the transmission pattern of behavior in anonymous social networks. In contrast to direct and indirect reciprocal behaviors, the enlarged influence of intention in generalized reciprocity may contribute to interventional strategies to reduce the spread of negative behavior within the general public while promoting prosocial interactions.

In light of how previous intention information affects the following interaction with other strangers, we speculate that emotion might play a mediating role. On the one hand, intention information was found to manipulate emotions (e.g., van’t Wout et al., 2006; Yu et al., 2017). In a pain-sharing study, for instance, participants with other’s intentional help reported less painful and stronger gratitude emotion than those with unintentional help (Yu et al., 2017). On the other hand, previous research has shown that positive emotion, especially gratitude emotion, works crucially in reciprocal behavior. Bartlett and DeSteno (2006) measured participants’ emotional state during reciprocity tasks, showing that participants with others’ help rated higher in gratitude emotion than those without help did, as well as demonstrating a mediating effect of gratitude emotion in helping behavior delivery. Later research using a gambling task further supported these findings (DeSteno et al., 2010). Similarly, negative emotion from others’ greedy treatment also predicted the individual’s allocation decision in generalized reciprocity to a certain extent (Gray et al., 2014; Strang et al., 2016). Hence, the emotional state in previous interactions has been highlighted as an important source to induce generalized reciprocal behaviors (Nowak and Roch, 2007). Taken together, we infer that intention information in current studies might raise corresponding emotions and further reinforce the effect of behavior transmission. To elaborate, generous assignment with subjective intention might stimulate strong gratitude emotion, while greedy assignment with subjective intention might be accompanied by intense negative emotion, resulting in a rather evident transmission tendency. This speculation needs to be tested in future research.

Above all, the intrinsic attributes of generalized reciprocal behavior further deepen the applicability of the current results. As behavior propagates in the social network, the behavioral transmission chain would be extended to include three or more nodes, and the corresponding reciprocal effects would gradually weaken (Liu et al., 2015). It is therefore worth exploring the pattern and duration of the effects of intention manipulation, which would thus have a certain value in application. In addition, following previous research, we used a computer program to make the random allocation decision for the two players in the unintentional condition. Future studies should take into account the situation where an unrelated third person makes the decision instead of a program, so as to include the factor of animacy to enhance the robustness of the current findings. Beyond that, more game types, such as the Ultimatum Game (e.g., Miyaji et al., 2013), should be considered to simulate multiple real situations and reinforce the external reliability of current findings. Previous investigations of generalized reciprocity in consumption scenes outside the laboratory (Jung et al., 2014) might provide new insights into intention-related reciprocity issues in the future.

## Data Availability Statement

The datasets generated for this study are available on request to the corresponding author.

## Ethics Statement

The studies involving human participants were reviewed and approved by the Research Ethics Board of the Department of Psychology in Ningbo University. The patients/participants provided their written informed consent to participate in this study.

## Author Contributions

WY and ZS contributed conception and design of the study. CY and ZH performed the experiments. CY, ZS, and ZH performed the statistical analysis. ZS wrote the first draft of the manuscript. WY reviewed and improved the manuscript. All authors read and approved the submitted version.

## Conflict of Interest

The authors declare that the research was conducted in the absence of any commercial or financial relationships that could be construed as a potential conflict of interest.

## Footnotes

1 Some researchers had also defined the conventional indirect reciprocity and generalized reciprocity as downstream indirect reciprocity and upstream indirect reciprocity, respectively.

## References

[B1] Alvarez K. van Leeuwen E. (2015). Paying it forward: how helping others can reduce the psychological threat of receiving help. *J. Appl. Soc. Psychol.* 45 1–9. 10.1111/jasp.12270

[B2] Bartlett M. Y. DeSteno D. (2006). Gratitude and prosocial behavior: helping when it costs you. *Psychol. Sci.* 17 319–325. 10.1111/j.1467-9280.2006.01705.x 16623689

[B3] Chao M. (2018). Intentions-based reciprocity to monetary and non-monetary gifts. *Games* 9:74 10.3390/g9040074

[B4] Charness G. (2004). Attribution and reciprocity in an experimental labor market. *J. Labor Econ.* 22 665–688. 10.1086/383111

[B5] Cushman F. Sheketoff R. Wharton S. Carey S. (2013). The development of intent-based moral judgment. *Cognition* 127 6–21. 10.1016/j.cognition.2012.11.008 23318350

[B6] DeSteno D. Bartlett M. Y. Baumann J. Williams L. A. Dickens L. (2010). Gratitude as moral sentiment: emotion-guided cooperation in economic exchange. *Emotion* 10 289–293. 10.1037/a0017883 20364907

[B7] Dufwenberg M. Kirchsteiger G. (2004). A theory of sequential reciprocity. *Games Econ. Behav.* 47 268–298. 10.1016/j.geb.2003.06.003

[B8] Falk A. Fehr E. Fischbacher U. (2003). On the nature of fair behavior. *Econ. Inq.* 41 20–26. 10.1093/ei/41.1.20

[B9] Falk A. Fehr E. Fischbacher U. (2008). Testing theories of fairness - Intentions matter. *Games Econ. Behav.* 62 287–303. 10.1016/j.geb.2007.06.001

[B10] Falk A. Fischbacher U. (2006). A theory of reciprocity. *Games Econ. Behav.* 54 293–315. 10.1016/j.geb.2005.03.001

[B11] Gan T. Lu X. Li W. Gui D. Tang H. Mai X. (2016). Temporal dynamics of the integration of intention and outcome in harmful and helpful moral judgment. *Front. Psychol.* 6:22 10.3389/fpsyg.2015.02022 PMC4708004 26793144

[B12] Gray K. Ward A. F. Norton M. I. (2014). Paying it forward: generalized reciprocity and the limits of generosity. *J. Exp. Psychol. Gen.* 143 247–254. 10.1037/a0031047 23244034

[B13] Halali E. Kogut T. Ritov I. (2017). Reciprocating (more) specifically to you: the role of benefactor’s identifiability on direct and upstream reciprocity. *J. Behav. Decis. Mak.* 30 473–483. 10.1002/bdm.1966

[B14] Herne K. Lappalainen O. Kestilä-Kekkonen E. (2013). Experimental comparison of direct, general, and indirect reciprocity. *J. Sociol. Econ.* 45 38–46. 10.1016/j.socec.2013.04.003

[B15] Horita Y. Takezawa M. Kinjo T. Nakawake Y. Masuda N. (2016). Transient nature of cooperation by pay-it-forward reciprocity. *Sci. Rep.* 6:19471. 10.1038/srep19471 26786178 PMC4726336

[B16] Jung M. H. Nelson L. D. Gneezy A. Gneezy U. (2014). Paying more when paying for others. *J. Pers. Soc. Psychol.* 107 414–431. 10.1037/a0037345 25133724

[B17] Liu P. P. Safin V. Yang B. Luhmann C. C. (2015). Direct and indirect influence of altruistic behavior in a social network. *PLoS One* 10:e0140357. 10.1371/journal.pone.0140357 26469066 PMC4607358

[B18] Miyaji K. Wang Z. Tanimoto J. Hagishima A. Kokubo S. (2013). The evolution of fairness in the coevolutionary ultimatum games. *Chaos Solit. Fract.* 56 13–18. 10.1016/j.chaos.2013.05.007

[B19] Nowak M. A. Roch S. (2007). Upstream reciprocity and the evolution of gratitude. *Proc. R. Soc. B Biol. Sci.* 274 605–610. 10.1098/rspb.2006.0125 17254983 PMC2197219

[B20] Nowak M. A. Sigmund K. (2005). Evolution of indirect reciprocity. *Nature* 437 1291–1298. 10.1038/nature04131 16251955

[B21] Orhun A. Y. (2018). Perceived motives and reciprocity. *Games Econ. Behav.* 109 436–451. 10.1016/j.geb.2018.01.002

[B22] Pfeiffer T. Rutte C. Killingback T. Taborsky M. Bonhoeffer S. (2005). Evolution of cooperation by generalized reciprocity. *Proc. R. Soc. B Biol. Sci.* 272 1115–1120. 10.1098/rspb.2004.2988 16024372 PMC1559812

[B23] Rabin M. (1993). Incorporating fairness into game theory and economics. *Am. Econ. Rev.* 83 1281–1302. 10.2307/2117561

[B24] Rankin D. J. Taborsky M. (2009). Assortment and the evolution of generalized reciprocity. *Evolution* 63 1913–1922. 10.1111/j.1558-5646.2009.00656.x 19222566

[B25] Simpson B. Harrell A. Melamed D. Heiserman N. Negraia D. V. (2018). The roots of reciprocity: gratitude and reputation in generalized exchange systems. *Am. Sociol. Rev.* 83 88–110.

[B26] Stanca L. Bruni L. Mantovani M. (2011). The effect of motivations on social indirect reciprocity: an experimental analysis. *Appl. Econ. Lett.* 18 1709–1711. 10.1080/13504851.2011.560105

[B27] Strang S. Grote X. Kuss K. Park S. Q. Weber B. (2016). Generalized negative reciprocity in the dictator game: how to interrupt the chain of unfairness. *Sci. Rep.* 6:22316. 10.1038/srep22316 26924557 PMC4770415

[B28] Trivers R. L. (1971). The evolution of reciprocal altruism. *Q. Rev. Biol.* 46 35–57. 10.1086/406755

[B29] Vaish A. Hepach R. Tomasello M. (2018). The specificity of reciprocity: young children reciprocate more generously to those who intentionally benefit them. *J. Exp. Child Psychol.* 167 336–353. 10.1016/j.jecp.2017.11.005 29227851

[B30] van’t Wout M. Kahn R. S. Sanfey A. G. Aleman A. (2006). Affective state and decision-making in the ultimatum game. *Exp. Brain Res.* 169 564–568. 10.1007/s00221-006-0346-5 16489438

[B31] Watanabe T. Takezawa M. Nakawake Y. Kunimatsu A. Yamasue H. Nakamura M. (2014). Two distinct neural mechanisms underlying indirect reciprocity. *Proc. Natl. Acad. Sci. U.S.A.* 111 3990–3995. 10.1073/pnas.1318570111 24591599 PMC3964069

[B32] Yu H. Cai Q. Shen B. Gao X. Zhou X. (2017). Neural substrates and social consequences of interpersonal gratitude: intention matters. *Emotion* 17 589–601. 10.1037/emo0000258 27936814

[B33] Zitek E. M. Jordan A. H. Monin B. Leach F. R. (2010). Victim entitlement to behave selfishly. *J. Pers. Soc. Psychol.* 98 245–255. 10.1037/a0017168 20085398

